# Dietary Intake, Body Composition and Iron Status in Experienced and Elite Climbers

**DOI:** 10.3389/fnut.2020.00122

**Published:** 2020-08-05

**Authors:** Edward Gibson-Smith, Ryan Storey, Mayur Ranchordas

**Affiliations:** ^1^Centre for Sport and Exercise Science, College of Health, Wellbeing and Lifestyle, Sheffield Hallam University, Sheffield, United Kingdom; ^2^Sport Industry Research Centre, College of Health, Wellbeing and Lifestyle, Sheffield Hallam University, Sheffield, United Kingdom; ^3^Academy of Sport and Physical Activity, College of Health, Wellbeing and Lifestyle, Sheffield Hallam University, Sheffield, United Kingdom

**Keywords:** climbing, nutrition, bouldering, sport climbing, RED-S, energy availability, weight loss, sport

## Abstract

Climbing has developed into a popular recreational and elite sport, evidenced by a growing number of licenced competition athletes, and the acceptance into the Olympic calendar for Tokyo 2020. A nutritional assessment, including the evaluation of anthropometric and biochemical data, has not been previously reported in climbing athletes. Therefore, the aim of this study was to assess the dietary intake, body composition, and iron status in experienced climbers, across a range of performance levels. Forty climbers (n = 20 male, n = 20 female; 8.8 ± 6.6 years' experience; BMI 21.6 ± 1.7) aged 18–46 (30.3 ± 6.7 years) participated in the study. Dietary intake was recorded in a 3-days diet diary. Body composition was assessed using a skinfold profile and iron status via blood markers. Mean energy intake was 2154.6 ± 450 kcal·day^−1^, with 30% of male climbers and 5% of female climbers failing to meet predicted resting metabolic rate. Furthermore, 77.5% of participants failed to meet a predicted energy requirement to support a “moderate” training programme. There were no significant correlations between daily energy intake and exercise volume. Mean intake of carbohydrate, protein and fat was 3.7 ± 0.9 g·kg^−1^·day^−1^, 1.6 ± 0.5 g·kg^−1^·day^−1^, and 1.4 ± 0.4 g·kg^−1^·day^−1^, respectively, with no significant difference between genders. Approximately 17% of males (*n* =3) and 45% of females (*n* = 9) had a sub-optimal iron status. Thirty percent of females met the classification criteria for iron deficiency. Mean serum ferritin was significantly greater in males, compared to females (102.7 ± 54.9 vs. 51.4 ± 24.2 μg·L^−1^; *p* ≤ 0.01) and significantly lower in vegan/vegetarians vs. omnivores, in female climbers only (33.2 ± 14.8 vs. 57.5 ± 24 μg·L^−1^; *p* = 0.05). No significant differences were observed between climbing ability groups (intermediate-advanced/elite-higher elite) for body composition, dietary intake, or iron status, for males or females. These findings suggest that experienced climbers are at risk of energy restriction and iron deficiency, therefore, routine assessment of nutritional status is warranted. Future research should consider iron status in relation to energy availability and investigate additional factors which may predispose this population to iron deficiency, as well as the risk of relative energy deficiency in sport (RED-S).

## Introduction

Climbing was originally devised as a training method for mountaineering and has since developed into a popular recreational and elite sport with an estimated 25 million people participating regularly, and up to 1,500 people trying the sport for the first time every day in the USA alone (1). This is further evidenced by a growing number of licenced competition athletes (2,160) from 65 countries and the acceptance of climbing into the Olympic calendar for Tokyo 2020, with provisional selection for Paris 2024.

In recent decades, an increasing number of studies have been carried out to investigate the anthropometric (2–6), biomechanistic (7, 8), physiological (9–17), and psychological (18) factors contributing to successful climbing performance. Despite the development of climbing into an elite sport, studies investigating the nutritional requirements of climbing are scarce, and no study to date has assessed the dietary intake of adult female climbers, or biochemical markers of iron status.

Reporting on lead rock climbers, Watts et al. (19) published the first large-scale assessment of anthropometric data in elite climbers, presenting findings from 39 athletes who reached the semi-finals of an international competition. The athletes were found to be relatively small in stature (male ~1.78 m, female ~1.65 m) with a low body mass (male ~66.6 kg, female ~51.1 kg). Since this pioneering work, numerous studies have reported similar findings, with some presenting climbers as excessively lean (6), but more recent work reporting similar results as other weight-sensitive sports (3, 5, 12). Anecdotally, the importance of power to weight ratio is well-recognised by climbers, with many sharing the view that excess fat provides additional resistance during ascent which can harm performance; therefore, a very lean physique is usually favoured. However, research has failed to establish a significant link between body composition and climbing performance thus far (4, 12).

Methods to determine an athlete's nutritional intake from food, fluids and supplement include weighed/measured food records (typically 3–7 days), 24-h dietary recall or food frequency questionnaires (20). Whilst all methods are prone to under-reporting error (21), those that depend on retrospective self-reporting of intake (e.g., 24-h recall) are more susceptible to conscious or sub-conscious exclusion of foods consumed (22), whilst the prospective weighing of foods can increase accuracy (23). It is recommended that a complete nutritional assessment process should also include the evaluation of anthropometric and biochemical data, such as iron status (24).

To date, only three published studies have assessed dietary intake in climbers. Zapf et al. (25) found a mean energy intake of ~2,650 kcal, with 40% of climbers consuming <2,500 kcal, despite training for more than 2 h per day. Nutritional intake of potatoes and vegetables appeared to be alarmingly low, representing merely 1.8% of total energy intake. Kemmler et al. (26) found similar results, with mean intakes of ~2,670 kcal·day^−1^. The energy intake of the climbing athletes was not significantly greater than their BMI matched controls, despite a 9.5 times greater training volume (401 ± 73 min vs. 43 ± 25 min). This pattern also appears to be consistent in adolescent climbers. Michael et al. (27) reported a weak correlation between training volume and energy intake, with 82% of adolescent climbers failing to meet their target daily energy intake. The prevalence of disordered eating and/or eating disorders among athletes in weight-sensitive sports is greater than other sports in which leanness is a not a prioritised performance variable (28), however research in this area within climbing is currently lacking.

These findings (25–27) suggest that climbers are at risk of chronic energy restriction and low energy availability (LEA). Adequate energy intake is important for maintaining health, immunity and injury resilience, as well as growth and repair, and optimising sports performance (29). Furthermore, LEA may contribute to iron deficiency (30) which can attenuate muscle function and capacity, leading to impaired training adaptation and performance, with or without anaemia (31). Despite these negative physiological effects, iron deficiency is commonly reported in athlete populations, affecting ~15–35% of female and ~3–11% of male athletes (32). Potential factors proposed to impact an athlete's iron stores include vegetarian diets and endurance exercise (33). However, no study to date has investigated iron status in climbers.

A nutritional assessment, including the evaluation of anthropometric and biochemical data, has not been previously reported in climbing athletes. Furthermore, no previous research has assessed the dietary intake of female climbers. Therefore, the aim of this study was to assess the body composition, dietary intake, iron status and supplement use among experienced climbers, from the recreational to the elite.

## Methods

### Participants

Participants were recruited using social media, online climbing forums and posters in local climbing centres (Sheffield, UK). Forty climbers (20 females, 20 males) volunteered to participate. Participants were required to meet the following inclusion criteria: age ≥ 18 years, ≥2 years climbing experience, currently taking part in climbing or climbing specific training ≥2 × per week, in good health with no acute or chronic illness that may influence dietary intake.

### Questionnaire

Participants answered a series of questions to identify years of climbing experience, predominant climbing discipline (bouldering/sport), weekly training/climbing volume, dietary preference (vegan/vegetarian or omnivorous) and highest climbing difficulty grade attained in the last 6 months. Self-reported climbing ability has been shown to be a valid representation of actual climbing ability (34). Climbing grades were converted from Font (bouldering) and French/sport (sport climbing) grading systems to the International Rock Climbing Research Association Reporting Scale to support a common approach to the statistical analyses within rock climbing research (35).

### Body Composition

Body mass was measured to the nearest 0.1 kg using electronic scales (Tanita, Japan), and height to the nearest 0.1 cm using a wall-mounted stadiometer (Holtain Ltd., UK). Body composition was assessed using the International Society for the Advancement of Kinanthropometry (ISAK) 8-site skinfold profile (36), carried out by an ISAK certified (Level 1) practitioner with an average technical error of measurement of 1%. Skinfold thickness was measured to the nearest 0.2 mm at eight sites (biceps, triceps, subscapular, iliac crest, supraspinal, abdominal, anterior thigh, and medial calf) using research standard calipers (Harpenden, UK). Duplicate measures were taken at each site and, where the technical error of measurement (TEM) was <5%, the mean value was reported. Where the TEM was >5%, a third measure was taken, and the median value reported. Girth measurements (relaxed arm, waist, gluteal and calf) were taken using a Lufkin metallic tape (W606PM, ATG, US). Body fat and fat-free mass (FFM) percentages were calculated using the Durnin and Womersley (37) equation, which has previously been validated against dual-energy X-ray absorptiometry in elite sport climbers (38).

### Estimating Energy Needs

Resting metabolic rate (RMR) was calculated using the Cunningham (1980) equation [RMR (kcal·day^−1^) = 500 + 22 (Fat Free Mass)]. This equation was chosen for its established application in highly active individuals (39). The RMR for each climber was multiplied by a physical activity factor of 1.443 to represent a “moderate” exercise level. Due to the highly variable energy requirements of climbing, and lack of reliable predictive models, this calculation was used as a conservative estimate to allow comparisons to be made between the actual intake of the climbers, and the energy needs to support a “moderately active” training programme. The self-reported data collected suggested that all participants had a training volume that met or exceeded the criteria for a “moderate” exercise level (3 × hard/5 × light sessions per week).

### Dietary Assessment

Participants were instructed to weigh (in grams) or measure (in millilitres) all foods, fluids and supplements consumed in a 3-days non-consecutive diary, an accepted method used in previous research to collect this type of data (40). Measurements were taken using self-owned, commercially available digital kitchen scales or by reporting manufacturer weights. An electronic template was provided with guidance notes detailing the requirements for accurate reporting. Participants were instructed to choose three non-consecutive days within a 7-days period to record, and to consider capturing days with a range of training demands (e.g., rest day; indoor climbing training; outdoor climbing session). When consuming pre-packaged products, participants were required to report the brand name, the weight of the product and manufacturer nutritional values. The diet diaries were analysed by a registered sports nutritionist using Nutritics software (Version 5.096). Where exact foods were not listed within the software database, similar foods with matched macronutrient composition were selected. Dietary supplement intake was included within the dietary analysis, therefore, contributing to the energy, macro-, and micro-nutrient content of the log.

### Assessment of Iron Status

A random, non-fasted venepuncture blood sample was taken by a trained phlebotomist in line with the World Health Organisation guidelines on drawing blood (41). Blood sampling was not restricted to a specific time of the day in line with diurnal variation data in markers of iron status (42), with fasting conditions recommended only when assessing iron overload (43). Blood was collected in a serum vacutainer (BD, USA) for analysis of serum ferritin and transferrin saturation, and an EDTA vacutainer for follow up haemoglobin analysis. Serum samples were allowed to clot for 30–60 min at room temperature, then processed in a centrifuge for 10 min at ~2,000 × g (Clinispin Horizon 642E, Drucker Diagnostics, USA) before being securely packaged and posted, as per testing laboratory guidelines. Serum ferritin and transferrin saturation were analysed by an external nationally recognised medical laboratory (TDL, London, UK), using a Roche “Cobas 8000” blood analyser (Roche Diagnostics GmbH, Germany). Serum ferritin was analysed using the sandwich principle; iron using a colorimetric assay; and unsaturated iron binding capacity (UIBC) via direct determination with FerroZine. Transferrin saturation was calculated as; transferrin saturation = [(iron) × 100/(iron+UIBC)]. Haemoglobin was analysed immediately after blood collection using the azide-methemoglobin method (Hb 201 System; HemoCue AB, Sweden) in participants with sub-optimal iron status; serum ferritin (<35 μg·L^−1^) and/or transferrin saturation (<20%) (44, 45). The sub-optimal iron status cut-off points specified were the same for both genders.

### Statistical Analysis

Statistical analysis was performed using SPSS software (version 24, IBM, USA). Data was checked for homogeneity of variance using Levene's test, and normality using Shapiro-Wilk's. Non-conforming data sets were transformed using Log-10. The differences in variables between groups (e.g., males vs. females) was analysed using an independent samples *t*-test, with significance set at *p* ≤ 0.05. A Pearson correlation coefficient determined the relationship between data sets (e.g., ability and energy intake). Correlation values (*R*^2^) were set as <0.2: weak correlation, 0.5: medium correlation, and >0.8: strong correlation (46). Data are presented as means ± standard deviation (SD), unless otherwise stated.

## Results

### Participant Demographics

Participant demographics are shown in Table 1. Forty experienced climbers (*n* = 20 male, *n* = 20 female; 8.8 ± 6.6 years' experience) aged 18–46 (mean age 30.3 ± 6.7 years) participated in the study. The average climbing ability of the cohort using the International Rock Climbing Research Association (IRCRA) scale was 22.2 ± 3.7; 47.5% (*n* = 19) of the climbers were classed as intermediate to advanced level [IRCRA score: 10–23 for males, 10–20 for females; (35)], with 52.5% (*n* = 21) meeting classification criteria for elite or higher elite (IRCRA score: 24–32 for males, 21–32 for females (35). Twenty-seven climbers identified “bouldering” as their primary climbing discipline, with the remaining thirteen climbers reporting “sport climbing.” One climber reported a previous case of iron deficiency anaemia. No other known health issues, or the use of medications that could impact dietary intake were reported. Average BMI was 21.6 ± 1.7; a BMI of <18.5, defined as potentially “underweight” (47) was reported in one female participant. Over a quarter (27.5%) of the climbers reported being vegan (*n* = 5) or vegetarian (*n* = 6).

**Table 1:** Participant demographics.

	**Males**	**Females**
*n*	20	20
Age (years)	29.1 ± 5.4	31.4 ± 7.7
Height (cm)	177.1 ± 6.9	166.8 ± 4.7
Mass (kg)	69.4 ± 5.8	58.5 ± 5.7
BMI	22.1 ± 1.4	21.1 ± 1.8
Experience (years)	7.8 ± 4.6	9.7 ± 8.2
Climbing Volume (min)	391 ± 181	497 ± 228
Training Volume (min)	214 ± 178	347 ± 193
IRCRA score	23.4 ± 2.9	21.1 ± 4.1
**Ability (n)**		
Intermediate-Advanced	13	6
Elite-Higher Elite	7	14
**Discipline (n)**		
Bouldering	17	10
Sport	3	10
**Dietary preference (n)**		
Vegan/Vegetarian	6	5
Omnivore	14	15

*Numbers expressed as means ± SD*.

*Intermediate-Advanced = IRCRA score; 10–23 males, 10–20 females (35)*.

*Elite-Higher Elite = IRCRA score; 24–32 males, 21–32 females (35)*.

### Body Composition

Body composition results are shown in Table 2. With the exception of gluteal girth, statistical analysis revealed significant gender differences across all the measured parameters (*p* ≤ 0.05). No significant differences were observed between ability groups (intermediate/advanced vs. elite/higher elite) for males or females. However, weak to medium correlations were seen in males between the IRCRA ability score and body mass (*R*^2^ = 0.506, *p* = 0.02), and height (*R*^2^ = 0.478, *p* = 0.03).

**Table 2:** Anthropometric data.

	**Males (♂)**	**Females (♀)**	**♂ vs. ♀**
Height (cm)	177.1 ± 6.9	166.8 ± 4.7	*p =* <0.01^*^
Mass (kg)	69.4 ± 5.8	58.5 ± 5.7	*p =* <0.01^*^
BMI	22.1 ± 1.4	21.0 ± 1.8	*p =* 0.04^*^
Sum of 8 SF	57.0 ± 19.5	83.2 ± 23.0	*p =* <0.01^*^
Body fat %	12.0 ± 3.8	22.9 ± 3.8	*p =* <0.01^*^
Arm girth (cm)	30.5 ± 1.8	27.2 ± 1.8	*p =* <0.01^*^
Waist girth (cm)	76.3 ± 3.5	66.9 ± 3.3	*p =* <0.01^*^
Gluteal girth (cm)	91.8 ± 3.7	92.62 ± 5.0	*p =* 0.58
Calf girth (cm)	35.5 ± 1.8	34.1 ± 1.8	*p =* 0.02^*^

*Mean ± SD. Males n = 20; females n = 20*.

* *P ≤ 0.05 is considered significant*.

### Dietary Intake

#### Energy Intake and Energy Requirements

Energy intake results are shown in Table 3. Mean energy intake was 2154.6 ± 450 kcal·day^−1^ (41.4 ± 9 kcal·kgFFM^−1^·day^−1^) for genders combined, with 30% of male climbers (*n* = 6) and 5% of female climbers (*n* = 1) failing to meet predicted RMR values. The 6 males identified consumed energy intakes 8–492 kcal·day^−1^ lower than their respective predicted RMR values (mean energy intake 238.3 ± 171.2 kcal·day^−1^ < RMR), whilst the female participant highlighted consumed 92 kcal·day^−1^ lower than the predicted RMR value (RMR = 1,526 kcal·day^−1^). Furthermore, 77.5% of climbers failed to meet a predicted energy requirement to support a “moderate” level of physical activity (Table 3). Females had a significantly higher energy intake than males when expressed relative to fat-free body mass (45.6 ± 7.0 vs. 37.2 ± 9.0 kcal·kgFFM^−1^·day^−1^; *p* ≤ 0.01).

**Table 3:** Dietary intake.

	**Males (♂)**	**Females (♀)**	**♂ vs. ♀**
**Predicted energy requirements**			
RMR (kcal·day^−1^)	1,842 ± 100	1486.3 ± 90	-
‘Moderate' Energy Needs (kcal·day^−1^)	2640.1 ± 143.5	2130.2 ± 129.2	-
**Energy intake**			
Total kcal·day^−1^	2270.4 ± 562	2038.8 ± 266.7	-
kcal·kgFFM^−1^·day^−1^	37.2 ± 9.0	45.6 ± 7.0	*p* = <0.01^*^
**Carbohydrate intake**			
Total g·day^−1^	251.7 ± 61.1	220.5 ± 46.3	-
g·kg^−1^·day^−1^	3.7 ± 1.0	3.8 ± 0.9	*p* = 0.65
**Protein intake**			
Total g·day^−1^	109.5 ± 34.8	92.6 ± 29.6	-
g·kg^−1^·day^−1^	1.6 ± 0.5	1.6 ± 0.5	*p* = 0.86
**Fat intake**			
Total g·day^−1^	90.9 ± 29.6	84.0 ± 18.9	-
g·kg^−1^·day^−1^	1.3 ± 0.4	1.4 ± 0.3	*p* = 0.31

*Mean ± SD. Males n = 20; females n = 20*.

* *P ≤ 0.05 is considered significant*.

**Figure 1:**
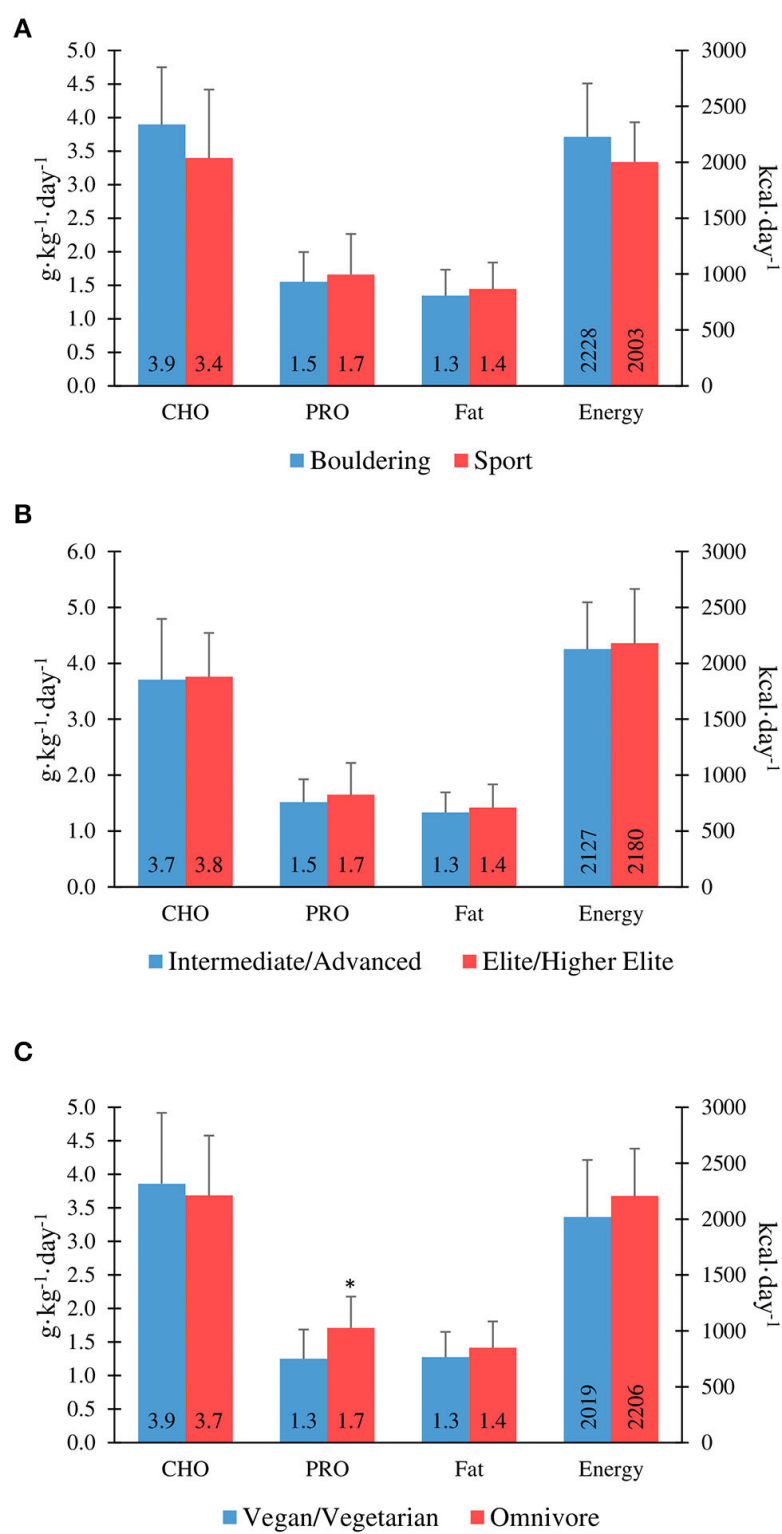
Macronutrient and energy intake in boulderers vs. sport climbers **(A)**, intermediate/advanced vs. elite/higher-elite **(B)**, and vegans/vegetarians vs. omnivores **(C)**. *Protein intake is significantly lower in vegan/vegetarians vs. omnivores (*P* ≤ 0.05).

There was no significant correlation between the IRCRA ability scale and energy intake (kcal·kgFFM^−1^·day^−1^) for males (*R*^2^ = −0.480, *p* = 0.84), or females (*R*^2^ = 0.201, *p* = 0.396). Furthermore, there were no significant correlations between total daily energy intake (kcal·day^−1^) and climbing or training volume, with *R*^2^ values of −0.246 (*p* = 0.13) and –0.005 (*p* = 0.97), respectively. Figure 1 shows energy intake comparisons between climbing discipline, ability, and dietary preference groups.

#### Macronutrient Intake

Macronutrient intake results are shown in Table 3. Mean intake of carbohydrate, protein and fat was 3.7 ± 0.9 g·kg^−1^·day^−1^, 1.6 ± 0.5 g·kg^−1^·day^−1^, and 1.4 ± 0.4 g·kg^−1^·day^−1^, respectively; with no significant difference between genders when expressed relative to body mass. One male participant reported a very low protein intake of 0.7 g·kg^−1^·day^−1^, failing to meet the dietary reference intake (DRI) for the general population [0.8 g·kg^−1^; (48)].

There were no significant differences between intermediate/advanced and elite/higher-elite level climbers for carbohydrate intake (3.7 ± 1.1 vs. 3.8 ± 0.8 g·kg^−1^·day^−1^; *p* = 0.86), protein intake (1.5 ± 0.4 vs. 1.7 ± 0.6 g·kg^−1^·day^−1^; *p* = 0.40), or fat intake (1.3 ± 0.4 vs. 1.4 ± 0.4 g·kg^−1^·day^−1^; *p* = 0.50) for genders combined (Figure 1). However, there was a significant correlation between the IRCRA ability scale and protein intake (g·kg^−1^·day^−1^) in female climbers (*R*^2^ = 0.452, *p* = 0.045). Only 17.5% of the cohort tested reported alcohol consumption.

Protein intake was significantly lower in vegan/vegetarians when compared to omnivores (1.25 ± 0.43 vs. 1.71 ± 0.47 g·kg^−1^·day^−1^; *p* = 0.007). There was no significant difference in protein intake between vegan and vegetarian climbers, when analysed separately (1.35 ± 0.49 vs. 1.17 ± 0.41 g·kg^−1^·day^−1^; *p* = 0.50). Overall daily protein intake was significantly higher in participants who used a protein supplement (*n* = 11; 2.0 ± 0.58 vs. 1.46 ± 0.40 g·kg^−1^·day^−1^; *p* ≤ 0.01). Figure 1 shows macronutrient intake comparisons between climbing discipline, ability, and dietary preference groups.

#### Iron Intake

**Figure 2:**
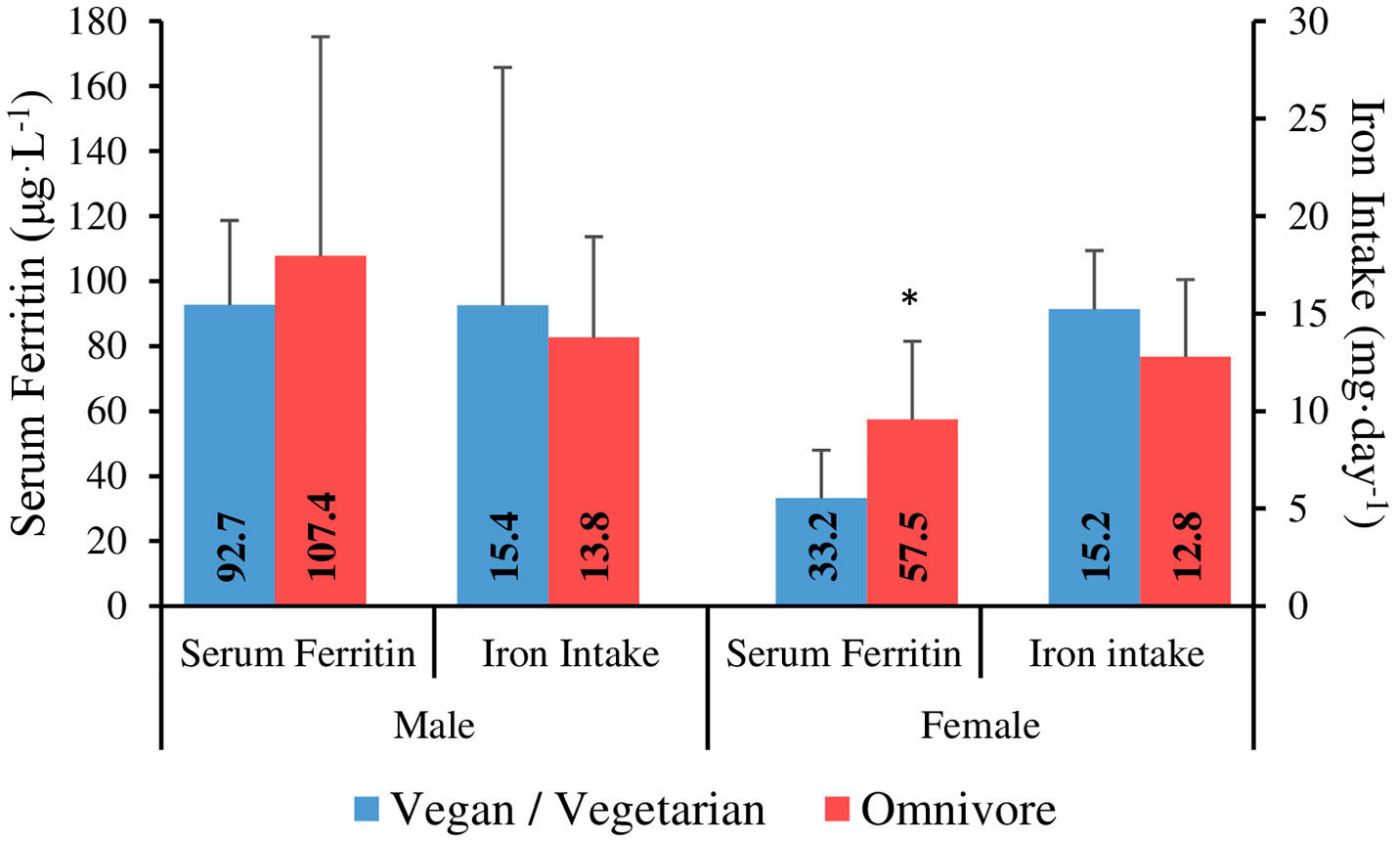
Serum ferritin and iron intake in vegan/vegetarian vs. omnivore climbers. *Serum ferritin is significantly lower in vegan/vegetarians vs. omnivores in females (*P* ≤ 0.05).

Iron intake results are shown in Table 4. Data from one male participant was omitted from the iron intake analysis due to taking a high dose iron supplement. Mean iron intake was 13.7 ± 6 mg·day^−1^, with no significant difference between gender groups (*p* = 0.66). Four male participants (~21%) and 16 female participants (80%) failed to meet the DRI for the general population [8 mg for males, 18 mg for females; (49)]. There was no significant correlation between iron intake (mg·day^−1^) and serum ferritin (μg·L^−1^) for males or females. There was a significant, medium strength correlation between iron intake (mg·day^−1^) and daily energy intake (*R*^2^ = 0.530, *p* = 0.001). Iron intake comparisons between genders and dietary preference groups are shown in Figure 2. There was no significant difference between vegan and vegetarian climbers in daily iron intake (mg·day^−1^) or iron intake density (mg·1,000 kcal^−1^·day^−1^) (*p* = 0.13; *p* = 0.09).

**Table 4:** Iron status.

	**Males (♂)**	**Females (♀)**	**♂ vs. ♀**
Serum ferritin (μg·L^−1^)	102.7 ± 54.9	51.4 ± 24.2	*p* = <0.01^*^
Transferrin saturation (%)	30.9 ± 15.9	26.7 ± 11.4	*p* = 0.39
Iron intake (mg)	14.1 ± 7.7	13.4 ± 3.8	*p* = 0.72
Iron intake density (mg·1,000 kcal^−1^)	5.95 ± 2.42	6.58 ± 1.71	*p = 0.35*

*Mean ± SD. Male serum ferritin n = 18, Male iron intake n = 19; females n = 20*.

* *P ≤ 0.05 is considered significant*. *Sub-optimal iron status; serum ferritin (<35 μg·L^−1^) and/or transferrin saturation (<20%) (44, 45)*.

#### Supplement Use

Forty-five percent of the climbers (males *n* = 10, females *n* = 8) recorded the use of one or more supplements. The most commonly used supplements reported were protein powder (*n* = 11), vitamin D (*n* = 7), multivitamins (*n* = 5) and fish oil capsules (*n* = 3). Other supplements reported (*n* ≤ 2) included creatine, beta-alanine, probiotics, vitamin C, turmeric, calcium, cissus, BCAA, glycine, collagen, vitamin B12, vitamin K, aloe vera, and meal replacements. The prevalence of supplement use was higher in intermediate/advanced level climbers (57.9%) compared to elite/higher-elite level (38.1%), whereas, the prevalence of supplement use amongst vegan/vegetarian climbers was comparable to omnivores (36.4 vs. 35.9%). Overall protein intake was ~31% higher in participants who used a protein powder supplement (124 ± 30.9 vs. 94.4 ± 31.0 g·day^−1^; *p* = 0.016).

### Iron Status

Iron status results are shown in Table 4. Data from two male participants was omitted from the iron status analysis. The aforementioned participant was excluded due to taking a high dose iron supplement, while hemochromatosis was incidentally identified in one participant during the study (serum ferritin 515 μg·L^−1^).

Sub-optimal iron status was found in 16.6% of males (*n* = 3) and 45% of females (*n* = 9). One quarter of females (*n* = 5) met the criteria for stage 1 iron deficiency (ferritin <35 μg·L^−1^, Hb > 115 g·L^−1^, transferrin saturation > 16% (44), and one female was identified with Stage 2 iron-deficient non-anaemia (ferritin <20 μg·L^−1^, Hb > 115 g·L^−1^, transferrin saturation <16%). Follow-up testing revealed only one male participant with anaemia (Hb <130 g·L^−1^).

There was no significant correlation between serum ferritin and energy intake (kcal·kgFFM^−1^·day^−1^) for males (*R*^2^ = −0.075, *p* = 0.77), or females (*R*^2^ = 0.05, *p* = 0.83). Furthermore, there were no significant differences between intermediate/advanced and elite/higher-elite level climbers for serum ferritin in males (111.7 ± 59.2 vs. 70.3 ± 25 μg·L^−1^; *p* = 0.08), or females (46 ± 28.2 vs. 53.7 ± 23.1 μg·L^−1^; *p* = 0.42). When analysed separately, there was no significant difference between vegan and vegetarian climbers for serum ferritin in females (30.7 ± 11.1 vs. 37.0 ± 24 μg·L^−1^; *p* = 0.82). However, serum ferritin was significantly lower in vegan/vegetarians combined when compared to omnivores in female climbers (33.2 ± 14.8 vs. 57.5 ± 24 μg·L^−1^; *p* = 0.05) (Figure 2).

## Discussion

This is the first study to perform a nutritional assessment, including the evaluation of anthropometric and biochemical data, in experienced male and female climbers across a range of abilities.

### Body Composition

Anthropometric and body composition data in the present study were similar to those previously reported in the literature (3, 5, 12, 38), with mean values for height, mass, BMI and body fat % of 177 cm, 69.4 kg, 22.1 (BMI), and 12.0% for males, and 166.8 cm, 58.5 kg, 22 (BMI), and 22.9% for females, respectively. Early research presented climbing athletes as short in stature, with a low body mass (19). Conversely, the present study shows significant weak to medium correlations in males between the IRCRA ability score and body mass, as well as height. However, it is difficult to draw conclusions as the existing anthropometric data in climbing research varies considerably, particularly body fat %, with a range of mean values reported between ~5–13% for males, and ~10–25% for females (3, 5, 12, 19, 38). Explanations for this variation might include the method used to assess body composition [Skinfold vs. DEXA; (38)], ability level, whether the climbers climb/compete indoors or exclusively climb outdoors, the timing of the measurement in relation to peak conditioning (particularly relevant to competition athletes), and the changing demands of the sport as it has developed over the years. Nevertheless, this study adds further data in climbers, where recent body composition figures are lacking.

There were no significant differences found in any of the anthropometric characteristics between ability groups for males or females, which again supports previous findings (5, 50). Furthermore, Laffaye et al. (2) determined that the only significant anthropometric difference found between novice and elite boulderers was an increased ape index, concluding that anthropometric variables explained only 4% of performance variation. Similarly, Mermier et al. (12) concluded that when anthropometric characteristics are similar, climbing performance is determined to a greater degree by training variables, rather than physique (58.9 vs. 0.3% of total variance). Considering the large variation in the physiological requirements of each climbing route or boulder problem, we have previously proposed that an ideal physique may not exist in climbing (51). Due to the reduced load and friction requirements, a lower mass may be favoured on a route with small holds and where there is more time spent static. Conversely, on routes with higher friction and more explosive, strength reliant moves, an athlete might benefit from greater muscle hypertrophy and consequently, increased force development (51).

### Dietary Intake

#### Energy Intake and Energy Requirements

Mean energy intake was ~2,270 kcal·day^−1^ for males, and ~2,039 kcal·day^−1^ for females, which is lower than the values previously reported (~2,650–2,670 kcal·day^−1^), however, this research either exclusively studied males (26), or did not clearly state which genders were assessed (abstract only; 26). Therefore, data reported in the present study potentially represents the first of its kind in adult female climbers. Concerningly, seven participants (six males, one female) failed to meet predicted resting metabolic rate (RMR), with 77.5% of climbers overall failing to meet a predicted energy requirement to support a “moderate” level of physical activity, despite a combined mean climbing and training volume of >12 h per week. Furthermore, there were no significant correlations between total daily energy intake (kcal·day^−1^) and climbing/training volume, in males or females. Under-reporting, either intentionally or unintentionally, is problematic using all methods of dietary assessment (52). Prevalence of underreporting in athletes is high, particularly in those who need to maintain a lean physique, such as gymnasts, with up to 61% classified as under-reporters (53). However, the participants in the present study were coached closely and an electronic template was provided with strict guidance notes detailing the requirements for accurate reporting, in an attempt to negate the effects of underreporting. Moreover, the data presented is consistent with previous research. Zapf et al. (25) reported that 40% of climbers failed to meet a conservative estimate of energy requirements (2,500 kcal·day^−1^), despite training for more than 2 h per day. Kemmler et al. (26) found similar results, reporting that the energy intake of the climbing athletes was not significantly greater than their BMI matched controls, despite a 9.5 times greater training volume. These findings also appear to be consistent in adolescent climbers, where a high prevalence of sub-optimal energy intake (82%) has been reported, with no significant association between training hours and energy intake (27). Furthermore, no difference was reported in energy intake between climbing ability groups (27), reflecting the data in the present study, where no significant correlation between ability and energy intake was seen in adult climbers. Despite a lower absolute energy intake compared to males (~2,039 vs. ~2,270 kcal·day^−1^), females had a significantly higher energy intake when expressed relative to fat-free body mass (45.6 vs. 37.2 kcal·kgFFM^−1^·day^−1^; *p* ≤ 0.01). This finding is likely due to the lower FFM and absolute body mass values reported in the female participants, when compared to the males.

It should be noted that the use of predictive equations to determine RMR values is a limitation of the present study and previous work in this field (27), due to the potential to generate an under or over prediction of energy requirements (39). Although the inclusion of body composition data may improve the accuracy of predictive equations in athletes (39), future research should consider a more precise method, such as indirect calorimetry (54).

A sufficient energy intake supports the optimal functioning of the body, determines the capacity for macronutrient and micronutrient intake, and influences body composition (29). Although gaining a reliable representation of energy intake using self-reporting methods is challenging (55), based on the data currently available it would be reasonable to suggest that climbers are at risk of energy restriction and/or low energy availability, evidenced by sub-optimal energy intakes, and a lack of adjustment of energy intake in relation to exercise volume.

Relative Energy Deficiency in Sport (RED-S) is a term which describes the myriad of consequences of consuming insufficient energy to meet the requirements for optimal physiological function in athletic populations (28). The negative health consequences of RED-S can be long lasting, and can impact menstrual function, bone health, metabolic, cardiovascular, endocrine, gastrointestinal, and immunological systems, as well as psychological well-being (28). Furthermore, RED-S may negatively impact athletic performance by impairing strength, endurance, injury risk, training response, coordination, concentration, and judgement (28). It is important to note that low energy availability and the development of RED-S can occur in the absence of weight loss, therefore it cannot be considered synonymous with energy balance (28).

#### Macronutrient Intake

There were no between-gender differences found for intake of carbohydrate, protein, or fat when expressed relative to body mass, which is in agreement with previous research (27, 56). No differences in macronutrient intake were found between intermediate/advanced and elite/higher-elite climbers in males or combined genders, in agreement with the findings of Sas-Nowosielski and Judyta (56), who also found no difference between ability levels. However, there was a significant correlation between climbing ability and protein intake in female athletes, supporting the work of Michael et al. (27), who found that elite climbers consumed more protein than intermediate climbers, although reported differences were small (1.8 vs. 1.7 g·kg^−1^·day^−1^).

Mean carbohydrate intake of 3.7 g·kg^−1^·day^−1^was similar to values previously reported (27, 56) and within the suggested range of 3–7 g·kg^−1^·day^−1^for climbers (57), although very much toward the lower end of the scale. Previous research that has attempted to provide guidelines on carbohydrate intake has primarily relied on extrapolations from other sports, so actual requirements are relatively unknown. Indeed, Tipton et al. (58) suggested that an intake of 5 g·kg^−1^·day^−1^ was necessary to prevent depletion of glycogen in sports that feature intermittent bouts of high-intensity resistance exercise.

Mean protein intake of 1.6 g·kg^−1^·day^−1^ was similar to values previously reported (27, 56) and double the recommendations for the general public [0.8 g·kg^−1^·day^−1^; (48)], indicating that climbers have an awareness of the necessity of protein for muscle remodelling and repair. As anticipated, the overall daily protein intake was ~31% higher in climbers who used a protein powder supplement (*p* = 0.016). Furthermore, when comparing protein intake relative to body mass in protein supplement non-users vs. users, mean values (1.46 ± 0.40 vs. 2.0 ± 0.58 g·kg^−1^·day^−1^; *p* ≤ 0.01) show a span similar to the proposed recommended range [1.3–2.0 g·kg^−1^·day^−1^; (51, 57)], suggesting a role of supplementation in climbers achieving the upper limits of recommended intake. Protein intake of vegan/vegetarian athletes was significantly lower than that of omnivores (1.25 vs. 1.71 g·kg^−1^·day^−1^; *p* = 0.007), and falls below the proposed recommendations for the sport [1.3–2.0 g·kg^−1^·day^−1^; (51, 57)]. This is in agreement with the findings of Clarys et al. (59) who found vegan/vegetarian diets to be generally lower in protein. Furthermore, plant-based proteins are generally regarded as being of lower quality than animal-based ones, as they are usually lacking one or more essential amino acids (60) and therefore, need to be carefully combined in order to meet the full spectrum of amino acids required. These findings suggest that vegan/vegetarian athletes may be at greater risk of protein insufficiency and may need to adopt targeted strategies to ensure that recovery, body-composition, and performance are not compromised.

#### Iron Intake

Mean iron intake was ~14 mg·day^−1^, with no significant difference between genders, however, 79% of males met the DRI for the general population, compared to just 20% of the females. This is primarily due to a large difference in proposed iron requirements (8 mg males, 18 mg females; 46), to compensate for menstrual losses (61).

Sim et al. (32) suggested that reducing energy intake may result in proportionally lower dietary iron consumption. The current findings are in agreement, with medium strength correlations found between iron intake and daily energy intake. Furthermore, this may offer some explanation as to why over 50% of climbers did not meet recommended iron intake, considering that 77.5% failed to consume enough energy to support “moderate” levels of exercise.

No correlation between iron intake and serum ferritin was found in the present study. A contributing factor could be the time-frame of data collection, as an athlete's dietary intake data over a 5–8 days period is recommended to provide an accurate assessment of micronutrient intake (24). However, collecting food records for longer than 3–4 days has been shown to reduce compliance and accuracy, as well as contribute toward a high drop-out rate (62). To increase the timeframe and size of data collection, short dietary records repeated several times, 2–3 months apart over different seasons, using non-consecutive random days, could be used in future research (63).

#### Supplement Use

Only one study to date has investigated supplement use amongst climbers (56), therefore, comparable data is limited. The prevalence of supplement use was relatively low (45%), considering that prevalence in other athletic populations has been reported around 81–100% (64). One contributing factor could be that the supplement data in this study was taken from food diaries alone, as opposed to a specific supplement history survey often used in other studies (65). Furthermore, tea and coffee were not included, despite containing caffeine, as intake was not assessed relative to training or competition. The most commonly used supplement was protein powder, supporting the findings of Sas-Nowosielski and Judyta (56), and appeared to increase the overall daily intake of protein. Supplementation was found to be more prevalent in intermediate/advanced climbers than in elite/higher elite. This was an unexpected finding considering that supplement use is generally higher in elite athletes than in their non-elite counterparts (65). The use of supplements in climbing athletes requires further investigation.

### Iron Status

Iron deficiency has been shown to negatively impact aerobic power, with larger deficiencies correlating with greater reductions in oxygen transport to the working muscles (32). Reduced aerobic power is likely to place greater demands on anaerobic metabolism during climbing (66), especially on steeper routes where it is considered the predominant energy source (9, 67), and may exacerbate decrements in performance. Despite resulting in impaired physiological function, iron deficiency is a commonly reported issue in athlete populations, affecting ~3–11% of male athletes, with a higher prevalence of ~15–35% seen in females (32). In the present study, 31.6% of participants had a sub-optimal iron status as defined in the literature (44, 45). The prevalence of sub-optimal iron status was greater in females (45%) compared to males (~17%), with one quarter of females meeting the criteria for stage 1 iron deficiency (ferritin <35 μg·L^−1^, Hb > 115 g·L^−1^, transferrin saturation > 16%) (44).

It has been suggested that low energy availability (LEA) may be partially induced by and result in the development of iron deficiency (30). The proposed mechanism is related to the iron deficiency induced perturbation of thyroid function, leading to decreased appetite and impaired metabolic efficiency; which can result in a reduced energy intake, increased energy expenditure and potentially, further exacerbation of LEA in athletes (30).

Considering the high prevalence of both sub-optimal energy intake and iron status in the climbing population assessed, it would be reasonable to suggest an interaction of this nature, however, statistical analysis revealed that there was no significant correlation between serum ferritin and energy intake for males, or females. Therefore, further research should consider iron status in relation to energy availability, rather than overall intake. Another factor to consider in the incidence of ID in this population is exercise-induced haemolysis, as previous research has suggested that this is exacerbated by muscle damaging exercise (68), and increased blood lactate levels (69); both of which are considered physiological features of climbing and training for climbing (70). Finally, menstrual blood losses of iron in females, may contribute toward the higher prevalence of ID seen within this population group (61). Larsson et al. (71) reported lower menstrual blood loss (~50%) in combined oral contraceptive pill (OCP) users compared to non-users, with OCP administration increasing serum ferritin levels by ~21–29% in subjects with pre-existing low iron stores (ferritin <10 μg·L^−1^), therefore, OCP use offers a therapeutic intervention for women who struggle to maintain iron stores due to heavy menstruation, with the potential additional benefit of protection from soft tissue injury (72). It is crucial to ensure that the dietary intake of the female athlete supports the energy needs of eumenorrhea and includes iron rich foods to attenuate a continued loss via menstruation. Future research may also consider controlling for variables which may affect iron loss in females.

Although there was no significant difference in iron intake between the dietary preference groups, serum ferritin was significantly lower in vegan/vegetarians when compared to omnivores in female climbers (33.2 vs. 57.5 μg·L^−1^), with no difference seen between vegan vs. vegetarian climbers when analysed separately. This could be explained by the different mechanism by which heme iron from animal products is absorbed compared to non-heme iron derived from plants, resulting in more efficient absorption which is less affected by accompanying dietary factors, and therefore, significantly greater bioavailability (73, 74). Whilst the presence of vitamin C can enhance the absorption of non-heme iron, chemicals (polyphenols and phytates) and minerals (calcium) that are found in tea, coffee, whole grains, legumes and dairy products, can inhibit the absorption of non-heme iron within a meal (75) and therefore, the overall intake and timings of these foods should be carefully considered by vegan/vegetarian athletes at risk of ID.

Limitations in the assessment of iron status in this study may lead to confounding results. For example, as an acute state reactant, serum ferritin (SF) may be artificially raised in response to intense exercise (20). Although participants were instructed to avoid exercise in the hours preceding the lab visit, exclusion of this variable relies on strict adherence to instruction. In addition, research suggests that SF may be decreased during menstruation in female participants (76), which is affected by variables such as the menstrual cycle phase (61), the use of birth control (71), or amenorrhea; induced by menopause, or LEA (28). Due to a lack of control of these variables, it is not possible to exclude this interaction and therefore, the data should be considered as preliminary at this stage, although the defined cut off values for ID are consistent irrespective of these variables. Furthermore, C-reactive protein, a marker of inflammation, was not measured and therefore it cannot be ensured that SF was not confounded by infection, inflammation, or injury (77), however, all participants reported to be in good health at the time of testing.

## Conclusions

These findings suggest that experienced climbers with intermediate to higher elite abilities practice energy restriction and are at risk of low energy availability, evidenced by sub-optimal energy intakes, and a lack of adjustment of energy intake in relation to exercise volume, supporting previous research. Furthermore, the preliminary data presented suggests that there is a high prevalence of climbing athletes at risk of iron deficiency, particularly females, who through dietary restriction may struggle to meet the higher gender specific iron intake targets. In view of the limitations outlined, future research should consider iron status in relation to energy availability and investigate additional factors which may predispose this population to iron deficiency, as well as the risk of relative energy deficiency in sport. Routine assessment of nutritional status by a qualified sports dietitian or sports medicine doctor is recommended in this population, with subsequent dietary guidance that focuses on increasing dietary iron intake and periodised energy provision in high risk athletes.

## Data Availability Statement

The datasets generated for this study are available on request to the corresponding author.

## Ethics Statement

The studies involving human participants were reviewed and approved by Sheffield Hallam University Research Ethics Committee. The patients/participants provided their written informed consent to participate in this study.

## Author Contributions

EG-S designed the study, collected, analysed and interpreted the data and produced the manuscript. RS assisted with data collection, production of figures, and preparation of the manuscript. MR supervised the study, advising on all elements, and performed the final editing of the manuscript. All authors gave final approval on the manuscript.

## Conflict of Interest

The authors declare that the research was conducted in the absence of any commercial or financial relationships that could be construed as a potential conflict of interest.
